# A water-forming NADH oxidase from *Lactobacillus pentosus* suitable for the regeneration of synthetic biomimetic cofactors

**DOI:** 10.3389/fmicb.2015.00957

**Published:** 2015-09-16

**Authors:** Claudia Nowak, Barbara Beer, André Pick, Teresa Roth, Petra Lommes, Volker Sieber

**Affiliations:** Chair of Chemistry of Biogenic Resources, Straubing Centre of Science, Department Life Science Engineering, Technische Universität München, StraubingGermany

**Keywords:** cofactor regeneration, H_2_O-forming NADH oxidase, synthetic cofactors, biomimetic cofactors, *Lactobacillus pentosus*, flavin adenine dinucleotide, hydrogen peroxide

## Abstract

The cell-free biocatalytic production of fine chemicals by oxidoreductases has continuously grown over the past years. Since especially dehydrogenases depend on the stoichiometric use of nicotinamide pyridine cofactors, an integrated efficient recycling system is crucial to allow process operation under economic conditions. Lately, the variety of cofactors for biocatalysis was broadened by the utilization of totally synthetic and cheap biomimetics. Though, to date the regeneration has been limited to chemical or electrochemical methods. Here, we report an enzymatic recycling by the flavoprotein NADH-oxidase from *Lactobacillus pentosus* (*Lp*Nox). Since this enzyme has not been described before, we first characterized it in regard to its optimal reaction parameters. We found that the heterologously overexpressed enzyme only contained 13% FAD. *In vitro* loading of the enzyme with FAD, resulted in a higher specific activity towards its natural cofactor NADH as well as different nicotinamide derived biomimetics. Apart from the enzymatic recycling, which gives water as a by-product by transferring four electrons onto oxygen, unbound FAD can also catalyze the oxidation of biomimetic cofactors. Here a two electron process takes place yielding H_2_O_2_ instead. The enzymatic and chemical recycling was compared in regard to reaction kinetics for the natural and biomimetic cofactors. With *Lp*Nox and FAD, two recycling strategies for biomimetic cofactors are described with either water or hydrogen peroxide as by-product.

## Introduction

The cell-free biocatalytic production of fine chemicals by oxidoreductases has continuously grown over the past years (Matsuda et al., 2009). When enzymes such as dehydrogenases that depend on the stoichiometric use of nicotinamide pyridine cofactors are involved, an integrated efficient recycling system is crucial to allow process operation under economic conditions (Weckbecker et al., 2010; Tauber et al., 2011). For a further increase of the profitability the variety of available cofactors for biocatalysis was recently broadened by the utilization of totally synthetic and cheap biomimetics (Lutz et al., 2004; Ryan et al., 2008; Campbell et al., 2012; Paul et al., 2013). Hence, the cost for the cofactor in a cell-free process can be decreased enormously (Rollin et al., 2013). In most cases these biomimetic cofactors can easily be synthesized in one or two steps. First, the oxidized form is prepared within the reaction of nicotinamide and the corresponding alkyl halogenide. Subsequent reaction with sodium dithionite yields the reduced form of the derivative (Karrer and Stare, 1937; Mauzerall and Westheimer, 1955). Several biomimetics have been described in literature so far: 5-methyl-1,4-dihydro-*N*-benzylnicotinamide and 6-methyl-1,4-dihydro-*N*-benzylnicotinamide (Takeda et al., 1987; Hentall et al., 2001), *N*-methyl-1,4-dihydronicotinamide (MNAH) (Friedlos et al., 1992; Knox et al., 1995) and *N*-benzyl-1,4-dihydronicotinamide (BNAH) (Lutz et al., 2004; Ryan et al., 2008; Paul et al., 2013). Though, to date the regeneration of the oxidized biomimetic cofactors has been limited to chemical or electrochemical methods (Lutz et al., 2004; Ryan et al., 2008; Poizat et al., 2010; Kara et al., 2014). An enzymatic recycling system would have the advantage of being evolvable and thereby holds the potential of a most efficient recycling in the future. The main challenge researchers are facing at the moment lies in finding enzymes that have at least a minor activity with biomimetic cofactors. So far only a few examples are known from literature: Paul et al. (2013) showed that the enoate reductase from *Thermus scotoductus* could use BNAH to perform the asymmetric reduction of conjugated C=C double bonds. Another example is the hydroxylation of non-activated C–H bonds by cytochrome P450 BM-3 R966D/W1046S from *Bacillus megaterium* with BNAH instead of the natural cofactor 1,4-dihydro-nicotinamide adenine dinucleotide (NADH) or 1,4-dihydro-nicotinamide adenine dinucleotide phosphate (NADPH) (Ryan et al., 2008). Most of the enzymes described so far need a second cofactor like flavin adenine mononucleotide (FMN) or flavin adenine dinucleotide (FAD) to be active. These flavin derivatives act as a linker between substrate and nicotinamide pyridine cofactor and are supposed to facilitate the hydride transfer (Paul et al., 2014a). From this it can be concluded that also other enzymes carrying flavin adenine cofactors have the potential to accept biomimetic cofactors. In this context NADH oxidases, which are also flavo-enzymes that oxidize NAD(P)H to NAD(P)^+^ with simultaneous reduction of molecular oxygen to either hydrogen peroxide (H_2_O_2_) or water (H_2_O), have attracted interest as suitable candidates for an efficient NAD^+^ cofactor recycling. In coupled enzyme reactions the four-electron reduction to benign H_2_O is preferred over the two-electron reduction to H_2_O_2_, which, even in small amounts, can inactivate the enzymes of the production-regeneration cycle (Hernandez et al., 2012). The addition of catalase as a possible remedy increases the complexity of the system to the point where three enzymes have to be coupled and adjusted to their activity over time. Water-forming NADH oxidases are described from various *Cocci* and *Bacilli* species: *Enterococcus (Streptococcus) faecalis* (Schmidt et al., 1986), *Streptococcus pyogenes* (Gao et al., 2012), *Streptococcus mutans* (Higuchi et al., 1993), *Lactobacillus rhamnosus* (Zhang et al., 2012), *Lactobacillus brevis* (Geueke et al., 2003), *Lactococcus lactis* (Heux et al., 2006), *Lactobacillus sanfranciscensis* (Riebel et al., 2003) as well as from *Clostridium aminovalericum* (Kawasaki et al., 2004) and from the hyperthermophile *Thermococcus profundus* (Jia et al., 2008). The sequence analysis of H_2_O-forming NADH oxidases from different *Lactobacilli* reveals sequence identities with a putative NADH oxidase from *Lactobacillus pentosus* (*Lp*Nox) that range from 42.5% for the crystallographically defined NADH oxidases from *L. sanfranciscensis* (pdb identifier CDU2) (Lountos et al., 2006) to 46.3 and 87.2% for the NADH oxidases from *L. brevis* (Geueke et al., 2003) and *L. plantarum* (Park et al., 2011), respectively. In each case, the most highly conserved regions include the redox-active cysteine (Cys42 in all sequences) as well as the FAD and NAD(P)H binding domains (see Supplementary Figure S1). In the proposed reaction mechanism of H_2_O-forming NADH oxidases the first NADP(H) transfers electrons onto FAD. FADH_2_ reduces molecular oxygen to hydrogen peroxide, which is supposed to be trapped in the active site and motioned toward the thiolate moiety of Cys42. A nucleophilic attack of Cys42-S^-^ on H_2_O_2_ yields the first water molecule and generates the Cys42–SOH intermediate. The second NAD(P)H then reduces FAD, which transfers the electrons much faster onto Cys42–SOH than onto another oxygen. Thus, the sulfenic acid is converted back to the thiolate and the second water molecule is released.

**FIGURE 1:**
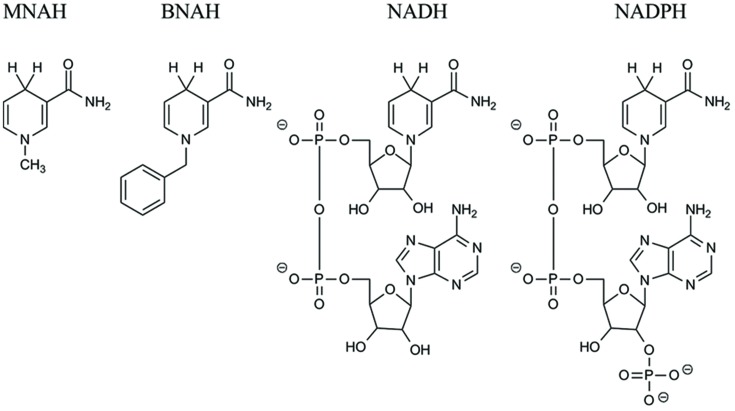
**Structural comparison of the biomimetics *N*-methyl-1,4-dihydronicotinamide (MNAH) and *N*-benzyl-1,4-dihydronicotinamide (BNAH), and the natural cofactors NAD(P)H**.

Due to the high sequence similarity to crystallographically defined H_2_O-forming NADH oxidases, we decided to investigate the putative NADH oxidase from *L. pentosus* MP-10 (*Lp*Nox) as a possible recycling enzyme in cell-free reactions. Furthermore, we tested the enzyme for the acceptance of the synthetic biomimetic cofactors MNAH and BNAH. The structures of all cofactors used are depicted in **Figure 1**. Interestingly, both *Lp*Nox and free FAD (Paul et al., 2014b, 2015) were capable of oxidizing the biomimetics and were therefore compared in regard of their kinetic parameters and the resultant by-products.

## Materials and Methods

### Reagents

All chemicals were purchased from Sigma–Aldrich, Merck or Carl Roth. All columns used for protein purification were from GE Healthcare (Munich, Germany).

### Synthesis of the Oxidized Biomimetics

The synthesis was adapted from a former procedure (Karrer and Stare, 1937; Mauzerall and Westheimer, 1955).

#### MNA^+^

Nicotinamide (50 mmol) was dissolved in 30 mL methanol. Methyl iodide (150 mmol) was added and the reaction mixture was stirred for 27 h at room temperature. The yellow precipitate was filtered and washed twice with methanol. The crude product was recrystallized from 250 mL hot methanol. C_7_H_9_IN_2_O; yellow solid; yield: 81%; ^1^H NMR (400 MHz, DMSO) δ 9.3 (s, 1H), 9.0 (d, 1H), 8.8 (d, 1H), 8.5 (s, 1H), 8.2 (dd, 1H), 8.1 (s, 1H), 4.4 (s, 3H).

#### BNA^+^

Nicotinamide (120 mmol) was dissolved in 40 mL acetonitrile and heated to reflux. Benzyl chloride (120 mmol) was added dropwise and the reaction mixture was stirred for further 12 h under reflux. After cooling to room temperature 120 mL diethyl ether was added, the precipitate was filtered and washed twice with diethyl ether. C_13_H_13_ClN_2_O; white solid; yield: 86%; ^1^H NMR (400 MHz, D_2_O) δ 9.2 (s, 1H), 8.9 (d, 1H), 8.8 (d, 1H), 8.1 (t, 1H), 7.4 (m, 5H), 5.8 (s, 2H).

### Synthesis of the Reduced Biomimetics

The synthesis was adapted from a former procedure (Karrer and Stare, 1937; Mauzerall and Westheimer, 1955; Knox et al., 2000).

#### MNAH

MNA^+^ (3.8 mmol) was dissolved in 250 mL water at 40°C under argon atmosphere. Sodium carbonate (24 mmol) and sodium bicarbonate (30 mmol) were added to the reaction mixture. Sodium dithionite (14 mmol) was added in portions and the mixture was stirred for further 30 min at 40°C. After cooling to room temperature an oily solid precipitated. The crude product was extracted three times with dichloromethane. The combined organic phases were washed once with water, dried over sodium sulfate and the solvent was removed by rotary evaporation. C_7_H_10_N_2_O; yellow solid; yield: 25%; ^1^H NMR (400 MHz, CDCl_3_) δ 7.0 (s, 1H), 5.7 (d, 1H), 5.4 (s, 2H), 4.7 (d, 1H), 3.1 (s, 2H), 2.9 (m, 3H).

#### BNAH

BNA^+^ (20 mmol) was added to a solution of sodium bicarbonate (66 mmol) and sodium dithionite (60 mmol) at 50°C and stirred for 15 min. The reaction mixture was cooled to 0°C for another 15 min. The precipitating solid was then washed twice with 100 mL dichloromethane and dried over sodium sulfate. The crude product was recrystallized from water:ethanol (3:1) at 0°C for 2 h. C_13_H_14_N_2_O; yellow solid; yield: 73%; ^1^H-NMR (400 MHz, CDCl_3_), δ (ppm): 7.34–7.23 (m, 5H), 7.15–7.14 (d, 1H), 5.74–5.73 (dq, 1H), 5.40 (br s, 2H), 4.76–4.72 (m, 1H), 4.28 (s, 2H), 3.17-3.16 (m, 2H).

### Cloning

For the cloning of the NADH oxidase (*Lp*Nox) from *L. pentosus* MP-10 (protein sequence GenBankTM CCB83530.1) genomic DNA was used as PCR template. It was isolated from cells of an overnight culture using the protocol of Chen and Kuo (1993). Three PCR reactions were performed to add an N-terminal, C-terminal or no His-Tag. For the N-terminal His-Tag the following primers were used: F-NheI-nox-*Lp* – GACAGGCTAGC**ATG**AAAGTTATCGTAATTGGTTGTAC and R-XhoI-stop-nox-*Lp* – GCGACTCGAG**TTA**TTCCGTCACTTTTTCAGCC, for the C-terminal His-Tag: F-BsaI-nox-*Lp*-CACGGTC TCGC**ATG**AAAGTTATCGTAATTGGTTGTAC and R-XhoI-nox-*Lp*-GCGACTCGAGTTCCGTCACTTTTTCAGCCGC and for no His-Tag: F-BsaI-nox-*Lp*-CACGGTCTCGC**ATG**AAAGTTATCGTAATTGGTTGTAC and R-XhoI-stop-nox-*Lp* – GCGACTCGAG**TTA**TTCCGTCACTTTTTCAGCC. The restriction enzyme recognition sides are underlined and the start and stop codon is marked in bold. The PCR products were purified and ligated into pET28a (Novagen, Darmstadt, Germany). The multiplication of the plasmid was performed with *E. coli* DH5α (Stratagene, La Jolla, CA, USA) in LB medium containing 30 μg/mL kanamycin.

### Expression and Purification

The expression of each variant of *Lp*Nox was performed with *E. coli* BL21 (DE3) in 200 mL autoinduction media with 100 μg/mL kanamycin (Studier, 2005). The preculture was incubated in 20 mL of LB medium with 30 μg/mL kanamycin at 37°C overnight on a rotary shaker (180 rpm). Expression cultures were inoculated with a 1:100 dilution of overnight cultures. Incubation was performed for 3 h at 37°C followed by incubation for 21 h at 16°C.

Subsequently, there was a separation in the handling for the His-tagged enzymes and the one without His-Tag. The treatment for both His-tagged enzymes was the same. Cells were harvested by centrifugation and resuspended in 50 mM sodium phosphate buffer (pH 8.0, 20 mM imidazole, 500 mM NaCl, and 10% glycerol). Crude extracts were prepared by the use of a Basic-Z Cell Disrupter (IUL Constant Systems), subsequent addition of MgCl_2_ to a final concentration of 2.5 mM in combination with DNase I (1 μg/mL) and a following incubation for 20 min at room temperature to degrade DNA. The insoluble fraction of the lysate was removed by centrifugation (20,000 rpm for 40 min at 4°C). The supernatant was filtered through a 0.45 μm syringe filter and applied to an IMAC affinity resin column, 5 mL HisTrapTM FF, equilibrated with the resuspension buffer using the ÄKTA Purifier-system. The column was washed with five column volumes of resuspension buffer and eluted in a gradient of 10 column volumes from 0 to 100% elution buffer (50 mM sodium phosphate buffer pH 8.0, 500 mM imidazole, 500 mM NaCl, and 10% glycerol). Aliquots of eluted fractions were subjected to 12% SDS-Page described by Laemmli (1970). The molecular weight of the His-tagged *Lp*Nox was calculated to be 51.94 kDa using the ProtParam tool (Artimo et al., 2012). The fractions containing the eluted protein were pooled and the protein was desalted using a HiPrepTM 26/10 Desalting column which was preliminary equilibrated with 50 mM Tris-HCl pH 7.5. Aliquots of the light yellow protein solution were frozen in liquid nitrogen and stored at -80°C.

Cells containing the enzyme without His-Tag were harvested by centrifugation and resuspended in 50 mM potassium phosphate buffer pH 7.0. Crude extracts were prepared by the use of a Basic-Z Cell Disrupter (IUL Constant Systems), subsequent addition of MgCl_2_ to a final concentration of 2.5 mM in combination with DNase I (1 μg/mL) and a following incubation for 20 min at room temperature to degrade DNA. The insoluble fraction of the lysate was removed by centrifugation (20,000 rpm for 40 min at 4°C). The supernatant was filtered through a 0.45 μm syringe filter and applied to an affinity resin column, 5 mL HiTrap Blue HP, equilibrated with the resuspension buffer using the ÄKTA Purifier-system. The column was washed with five column volumes of resuspension buffer and eluted in one step with elution buffer (50 mM potassium phosphate buffer pH 7.0 and 1.5 M KCl). Aliquots of the different fractions were subjected to 12% SDS-Page described by Laemmli (1970). The molecular weight was calculated to be 49.46 kDa.

### Determination of Protein and FAD Concentration

The *Lp*Nox concentration was determined using a Bradford assay Roti^®^-nanoquant (Carl Roth) with BSA as standard. The FAD concentration was measured in microtiter plates at 450 nm and compared to a FAD standard (10-70 μM).

### Enzyme Activation

The *Lp*Nox was incubated with an excess of FAD at 37°C for 15 min. After cooling the enzyme to 4°C precipitated protein was separated by centrifugation (20,000 rpm for 15 min at 4°C). Afterward unbound FAD was removed by size exclusion chromatography using a HiPrepTM 26/10 Desalting column, which had been preliminary equilibrated with 50 mM Tris-HCl pH 7.5. Aliquots of the protein were frozen in liquid nitrogen and stored at -80°C.

### General Characterization of *Lp*Nox

All enzyme assays were performed in triplicate with 50 mM buffer at the desired pH, concentration of NADH and at the desired temperature. The absorption of NADH was measured in microtiter plates (Greiner, flat bottom) at 340 nm using a Multiskan or Varioskan spectrophotometer (Thermo Fisher Scientific).

The pH activity profile was obtained at 25°C with 0.3 mM NADH in 50 mM triple buffer containing ⅓ sodium citrate, ⅓ potassium phosphate and ⅓ glycine. The pH values were adjusted with HCl or KOH from pH 5.0 to 10.0.

The optimum temperature was studied by adding the enzyme solution to the preheated reaction mixture containing 50 mM Tris-HCl pH 7.0 and 0.3 mM NADH. A temperature range of 25–50°C was chosen.

The thermostability (T_50_^30^) was studied by incubating the enzyme at various temperatures for 30 min in 50 mM Tris-HCl pH 7.0 with and without 5 mM DTT. The residual activity was measured at 37°C with 0.6 mM NADH.

The kinetic parameters of *Lp*Nox with NADH were investigated with a substrate range from 0.01 to 0.4 mM in 50 mM Tris-HCl pH 7.0. The calculation of Michaelis–Menten kinetics for determination of *K*_m_ and *v*_max_ was done with SigmaPlot 11.0 (Systat Software).

The total turnover number was calculated from *k*_cat_/*k*_deact_, whereas *k*_deact_ was obtained from incubating the enzyme at 37°C in 50 mM Tris-HCl pH 7.0 with and without 5 mM DTT. At certain time points the residual activity was measured with 0.3 mM NADH in the same buffer. The data points were fitted to an exponential decay equation using SigmaPlot 11.0 (Systat Software).

### Characterization of the *Lp*Nox with the Biomimetic Cofactors

Stock solutions of MNAH/BNAH were dissolved in DMSO freshly before use and added to the assay to give a final DMSO concentration of 5%.

The activity of the *Lp*Nox was measured in triplicates with 0.5 mM biomimetic in 50 mM Tris-HCl pH 7.0 at 37°C. The decrease of the absorbance of MNAH/BNAH was followed at 358 nm. The activity of an equivalent concentration FAD with the biomimetics was determined similarly.

The kinetic constants for the biomimetic cofactors were determined in triplicate with a fluorescence measurement. A solution of 20 μl 2 M potassium hydroxide, 20 μl 20% acetophenone in DMSO, and 45 μl H_2_O was prepared in a polypropylene fluorescence microtiter plate. The enzymatic reaction in 50 mM Tris-HCl pH 7.0 containing *Lp*Nox and substrate ranging from 0 to 8 mM was incubated at 37°C. Every 2–3 min a 5 μl sample from the enzymatic reaction was added to the solution. Then 90 μL 88% formic acid was added and it was incubated for 30 min at room temperature. The samples were measured at the following conditions: MNA^+^: excitation: 386 nm, emission: 446 nm; BNA^+^ excitation: 380 nm, emission: 438 nm (Zhang et al., 2011). For the calculation of concentrations a standard was used (0–0.8 mM oxidized cofactor). The calculation of Michaelis–Menten kinetics for determination of *K*_m_ and *v*_max_ was done with Sigma-Plot 11.0 (Systat Software).

### Quantification of H_2_O_2_

The H_2_O_2_ concentration was determined in a microtiter plate using 100 μL of the activity assay containing 1 mM MNAH/BNAH/NADH and 6 μM *Lp*Nox/FAD in 50 mM Tris-HCl pH 7.0, which was incubated for 5 h at 37°C, and 100 μL detection reagent containing 50 μM DA-64 and 0.2 U/mL horseradish peroxidase in 50 mM Tris-HCl pH 7.0. The absorbance was measured at 727 nm and the quantities were calculated by a standard curve (0–40 μM).

## Results

### Enzyme Purification and Activation with FAD

The attempt to use the HiTrap Blue HP column for purification of the untagged enzyme was not successful. The complete enzyme was found in the flow through. However, both enzymes with N-terminal or C-terminal His-Tag were soluble and could be purified as a yellowish solution due to the bound FAD. No major impurities were detected by SDS-PAGE (Supplementary Figure S2) and 84 mg of N-terminal *Lp*Nox were obtained from 1 L of culture. There was no significant difference in the catalytic activity of both tagged enzymes and therefore all experiments were conducted using the N-terminal His-Tag enzyme.

The activity of NADH oxidases depends on the cofactor FAD, which binds to the enzymes when it is translated and folded in the producing organism. We found that the overexpression in *E. coli* can result in poor activity of the *Lp*Nox due to missing FAD: 1 mole of purified *Lp*Nox contained between 0.12 and 0.2 mol of FAD. But the *Lp*Nox could be loaded with FAD, when the enzyme was incubated with an excess amount of free FAD. The incubation of 157 μM *Lp*Nox (containing 13% FAD) with 200 μM FAD at 37°C for 15 min and subsequent desalting to remove unbound FAD, resulted in 60 μM *Lp*Nox with 57 μM FAD (95%). The specific activity was 7.9 U/mg before activation and 50.1 U/mg after loading. Overall, a sevenfold improvement in FAD content and a 6 fold increase in specific activity were achieved.

### General Characterization

**FIGURE 2:**
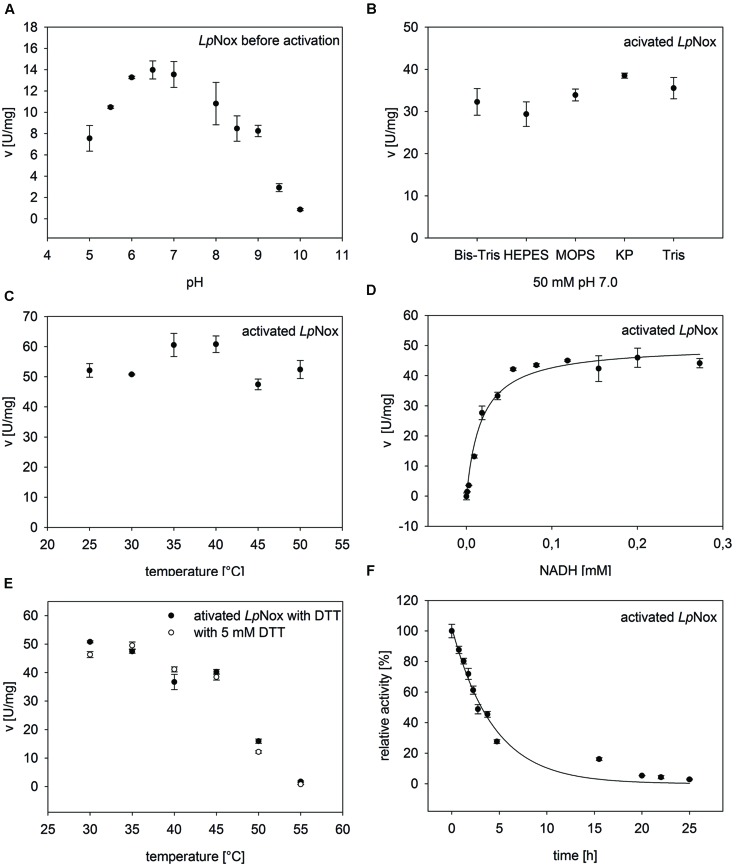
**General characteristics of Nox from *Lactobacillus pentosus* (*Lp*Nox).** *Lp*Nox activity was investigated in regard of **(A)** pH, **(B)** buffer type, and **(C)** temperature. **(D)** The kinetic parameters were determined by fitting the data of *Lp*Nox activity vs. NADH concentration to the Michaelis–Menten equation. **(E)** No influence of DTT on the thermal stability of *Lp*Nox was seen. **(F)** The half-life of *Lp*Nox at its temperature optimum (37°C) was determined by fitting the data points to an exponential decay equation. The error bars indicate the standard deviation of three measurements.

*Lp*Nox was investigated for its suitability as a regeneration enzyme. Therefore, it was tested under various conditions to determine influences on the activity (**Figure 2**).

#### pH, Buffer, and Temperature Effect

The pH profile showed a sharp peak at pH 7.0 with 70% residual activity at pH 6.0 and 8.0 (**Figure 2A**). The highest activity at pH 7.0 was measured in potassium phosphate buffer. But since potassium phosphate reduces cofactor stability (Rover et al., 1998), all further experiments were done in Tris-HCl pH 7.0 (92% activity compared to potassium phosphate; **Figure 2B**). The temperature optimum of *Lp*Nox was between 35 and 40°C. Overall, no strong temperature dependence could be detected during the first 5 min after starting the reaction (**Figure 2C**).

#### Kinetics and Reaction Product

From kinetic studies in 50 mM Tris-HCl pH 7.0 at 37°C, a *K*_m_ for NADH of 17.9 μM ± 3 μM and a *v*_max_ of 50.1 ± 1.9 U/mg (*k*_cat_ = 43.4 s^-1^) for NADH were determined (**Figure 2D**). NADPH can be recognized as a substrate by *Lp*Nox, though the specific activity was only about 2% compared to NADH. Due to a conserved Cys residue at position 42 *Lp*Nox should be an H_2_O-forming NADH oxidase. This hypothesis could be confirmed with an H_2_O_2_ assay, where less than 1% of the theoretical yield of hydrogen peroxide could be detected.

#### Thermal Stability and Total Turn Over

To determine the influence of temperature over a specific period of time, the thermal stability was tested by incubating the enzyme for 30 min at various temperatures and measuring the residual activity. The temperature, at which 50% of the activity was left compared to the activity before incubation (T_50_^30^), was 48.4°C. A similar result (48°C) was obtained when DTT was added to the enzyme during incubation (**Figure 2E**). The total turnover of an enzyme can be calculated from *k*_cat_/*k*_deact_. Therefore, the decrease in activity at 37°C until the enzyme was completely inactive was measured. The obtained values were fitted to an exponential function, giving a *k*_deact_ of 0.299 ± 0.0237 h^-1^ (**Figure 2F**). This resulted in a half-life of *Lp*Nox of 3 h and a total turnover number of 6.8*10^5^. In order to see whether the loss of activity over time was due to a loss of FAD, the incubated enzyme solution was filtered (10 kDa cutoff). The flow through did not contain FAD, whereas the residue on the filter was yellow.

### Activity with Biomimetic Cofactors

After the activation of the *Lp*Nox with FAD the activities with the biomimetic cofactors MNAH and BNAH could be increased by a factor of 4–6 (data not shown).

Recently, Paul et al. described the reaction of flavin mononucleotide (FMN) with the biomimetic cofactor BNAH under formation of H_2_O_2_ (Paul et al., 2014b, 2015). Accordingly, this chemical reaction was confirmed with FAD and the two biomimetics tested in this study (**Table 1**). In order to compare the enzymatic reaction to the reaction of free FAD, the turn over numbers (TON) were calculated with respect to the FAD content. With MNAH free FAD showed a slightly higher activity than the *Lp*Nox, while the activities with BNAH were equal. Since the *Lp*Nox is an FAD dependent enzyme the observed reaction could either be enzymatically or chemically catalyzed. To investigate this, the reaction products were determined. When incubating FAD with 1 mM of the biomimetic cofactors at 37°C for 5 h high quantities of H_2_O_2_ could be detected: 0.32 ± 0.040 mM for MNAH and 0.50 ± 0.018 mM for BNAH. In contrast, in the enzymatic reaction only small amounts of about 0.2*10^-3^ mM (MNAH) and 2.6*10^-3^ mM (BNAH) H_2_O_2_ were produced (**Table 2**). However, this could also be attributed to a possible catalase property of the *Lp*Nox. If *Lp*Nox is added after H_2_O_2_ production with free FAD, the concentration of hydrogen peroxide indeed decreases substantially but remains still significantly higher compared to the amount that was detected in the solely enzymatic reaction.

**Table 1:** Comparison of the turn over number (TON) of *Lp*Nox and FAD with the biomimetics.

Cofactor	TON of free FAD [min^-1^]	TON of *Lp*Nox [min^-1^]
MNAH	2.23 ± 0.15	1.31 ± 0.05
BNAH	0.97 ± 0.07	0.79 ± 0.01

*The activities of the enzymatic and chemical catalyst were investigated with the biomimetics MNAH and BNAH in a photometric assay (358 nm). The activities were compared concerning the FAD content. The standard deviation was calculated form three measurements.*

**Table 2:** Measurement of the by-product H_2_O_2_.

Cofactor	FAD [mM H_2_O_2_]	*Lp*Nox [mM H_2_O_2_]	FAD/*Lp*Nox [mM H_2_O_2_]
MNAH	0.32 ± 0.04	0.2*10^-3^± 0.1*10^-3^	16*10^-3^ ± 1*10^-3^
BNAH	0.51 ± 0.18	2.6*10^-3^ ± 0.9*10^-3^	25*10^-3^ ± 1*10^-3^

*H_2_O_2_ concentrations in the reactions of FAD or *Lp*Nox with 1 mM of the biomimetics MNAH and BNAH were detected with DA-64 and horseradish peroxidase. Firstly either FAD or the enzyme was added to the cofactor. Secondly a combined approach was performed with addition of *Lp*Nox after 1 h. The standard deviation was calculated from three measurements.*

**FIGURE 3:**
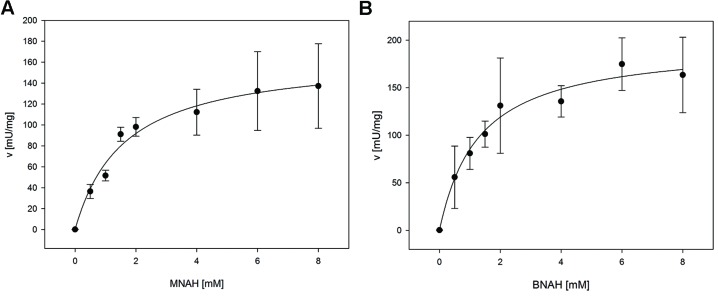
**Kinetic measurements of *Lp*Nox with biomimetic cofactors.** The kinetic parameters were determined by fitting the data of *Lp*Nox activity vs. cofactor concentration to the Michaelis–Menten equation (**A**, MNAH, **B**, BNAH). The error bars indicate the standard deviation of three measurements.

For the determination of the kinetic constants of *Lp*Nox with the biomimetic cofactors a fluorescence assay was used, because high concentrations of reduced cofactors exceed the maximal absorbance for photometric measurements. Both could be fitted to the Michaelis-Menten equation (**Figure 3**). *K*_m_ and *v*_max_ (*k*_cat_) of both cofactors are in the same range with BNAH showing a slightly lower *K*_m_ and higher *v*_max_ than MNAH. Therefore, *k*_cat_/*K*_m_ for BNAH is 1.4 higher than for MNAH (**Table 3**).

**Table 3:** Comparison of kinetic parameters of the biomimetics with *Lp*Nox.

Cofactor	*K*_m_ [mM]	*v*_max_ [mU/mg*_Lp_*_Nox_]	*k*_cat_ [s^-1^]	*k*_cat_/*K*_m_ [s^-1^ mM^-1^]
MNAH	1.6 ± 0.5	166.0 ± 16.9	0.14	0.09
BNAH	1.3 ± 0.4	198.9 ± 19.8	0.17	0.13

*The enzymatic catalysis with MNAH and BNAH was characterized with a fluorescence assay. The kinetic parameters were determined by fitting the data of *Lp*Nox activity vs. cofactor concentration to the Michaelis–Menten equation. The standard deviation was calculated from three measurements.*

## Discussion

### General Applicability of *Lp*Nox for the Regeneration of Oxidized Cofactors

With the NADH oxidase from *L. pentosus* (*Lp*Nox), we identified a regeneration system for the natural cofactor NAD^+^ as well as for the biomimetic cofactors MNA^+^ and BNA^+^. After expression and purification in *E. coli*, more than 80% of the enzyme was present in the apo-form and did not contain the essential cofactor FAD. Incubation of the enzyme with an excess amount of FAD and subsequent desalting resulted in fully activated *Lp*Nox that did not lose the FAD during desalting or the activity tests. This is similar to the procedure described by Jiang and Bommarius (2004) and Tóth et al. (2008) and obviates the need of adding FAD to the reaction mixture, which is also common (Jiang et al., 2005; Rocha-Martin et al., 2011). The disadvantage of the latter only becomes obvious when using the biomimetic cofactors, where this leads to the formation of hydrogen peroxide. The activated *Lp*Nox was investigated in regard of its possible application in cell-free reaction systems. Therefore, the effect of pH, buffers, temperature, the reducing agent DTT and the thermal stability were determined. The enzyme’s optimal activity range was from pH 5.5 to 8.0, a common range for NAD(P)H oxidases (Higuchi et al., 1993; Jiang et al., 2005; Park et al., 2011). The upper limit is compatible with most dehydrogenases, so appropriate coupling seems feasible. The amount of H_2_O_2_ released by *Lp*Nox during turnover is so low that it can be regarded as a water-forming NADH oxidase (Jiang et al., 2005; Lountos et al., 2006; Park et al., 2011). Deactivation of NADH oxidases through over oxidation of the catalytically active Cysteine residue has been described among others for the Nox of *L. brevis* and could be avoided by adding a reducing agent such as DTT (Hummel and Riebel, 2003). For the *Lp*Nox no influence of DTT on activity or total turnover number was seen, neither positive nor negative. Together with the low influence of the buffer type on activity, this makes *Lp*Nox a flexible enzyme for coupled reactions. Room for improvement lies in the specific activity of *Lp*Nox, which is rather low compared to the H_2_O-forming NADH oxidases from *Streptococcus pyogenes*: 344 U/mg (Gao et al., 2012) or *Lactobacillus brevis*: 116 U/mg (Hummel and Riebel, 2003). Also the rather low thermal stability at temperatures above 40°C could be improved by enzyme engineering.

Apart from NADH oxidases, an iron catalyzed oxidation of natural cofactors is possible (Maid et al., 2011). Here, also four electrons are transferred onto oxygen giving water as the by-product. Compared to the catalytic activity of *Lp*Nox, the metalloporphyrin is better in oxidizing NADPH (3.6 min^-1^), but worse in oxidizing NADH (6.6 min^-1^).

### Enzyme-Catalyzed vs. FAD-Catalyzed Regeneration of Biomimetic Cofactors

*Lp*Nox and FAD are both capable of oxidizing the biomimetic cofactors MNAH and BNAH. The use of the catalyst decides on which by-product is formed. The chemical catalyst FAD produces H_2_O_2_ during the oxidation of the biomimetic proposedly by a two hydride transfer. However, in coupled redox reactions H_2_O_2_ should be avoided, since it can damage the enzymes and substrates involved (Hernandez et al., 2012). The addition of a catalase would be possible, though this would add to the complexity of the system to the point where three catalysts (two enzymes and the chemical catalyst FAD) have to be matched. Therefore, using *Lp*Nox to regenerate biomimetic cofactors would be the superior choice over FAD, if it is optimized, e.g., by enzyme engineering. An exception is a case described by Paul et al. (2014b). Here the biomimetic cofactor BNAH plus FMN were used to specifically produce H_2_O_2_ *in situ* for the subsequent enzymatic reaction with a P450 peroxygenase. For this kind of application, both biomimetic cofactors tested in our study would to be suitable. Apart from the stated example, inhibitions in the biotransformation of interest by H_2_O_2_ can be avoided using the *Lp*Nox for cofactor regeneration instead. The conserved Cys residue at position 42 presumably acts as a second redox center. Therefore, in total a four electron transfer is achieved and innocuous water is produced. The kinetic experiments suggest that BNAH could be oxidized slightly more efficiently compared to MNAH. Because of the hydrophobic benzyl group in BNAH, the recognition and coordination in the cofactor binding site of *Lp*Nox is possibly better than for the smaller cofactor MNAH. Generally, the activity with free FAD is higher than with the enzyme. However, it has to be considered that the *Lp*Nox could be improved by enzyme engineering. If the interactions of the *Lp*Nox with the biomimetic cofactors could be increased, a more efficient catalysis might be obtained. In this way the amount of catalysts can be kept low while simultaneously the activity and effectivity is improved.

## Conclusion

In conclusion, we found a H_2_O-forming NADH oxidase from *L. pentosus* (*Lp*Nox) that is able to oxidize the natural cofactor NADH as well as the biomimetic cofactors MNAH and BNAH. The enzyme is highly active between pH 6.0 and 8.0, is independent of the buffer type and DTT, and has a temperature optimum of 35–40°C. The *K*_m_ for NADH is 17.9 μM and *k*_cat_ is 43.4 s^-1^, whereas for the biomimetic the *K*_m_ is 1.6 mM (1.3 mM) and *k*_cat_ is 0.14 s^-1^ (0.17 s^-1^) for MNAH (BNAH). Furthermore, we compared the reaction rates of the enzyme with the reaction rate of free FAD. Although, the *Lp*Nox has a lower or equal rate as free FAD, the reaction products differ. In case of *Lp*Nox innocuous water is formed, while with free FAD hydrogen peroxide is obtained. Considering the future application of biomimetic cofactors in cell-free coupled reaction systems with other enzymes, hydrogen peroxide would be hazardous and henceforth, the use of *Lp*Nox would be favorable. In order to enhance the activity of *Lp*Nox towards the synthetic cofactors, enzyme engineering could be performed.

## Conflict of Interest Statement

The authors declare that the research was conducted in the absence of any commercial or financial relationships that could be construed as a potential conflict of interest.
