# Supplementary material for: A water-forming NADH oxidase from Lactobacillus pentosus suitable for the regeneration of synthetic biomimetic cofactors
Source: Front Microbiol. 2015 Sep 16;6:957. doi: 10.3389/fmicb.2015.00957 (PMC4584968; doi:10.3389/fmicb.2015.00957)
Supplement: Supplementary file 1 [file Data_Sheet_1.DOCX]

***Supplementary Material***

**A water-forming NADH oxidase from *Lactobacillus pentosus* suitable for synthetic biomimetic cofactors**

**Claudia Nowak^1^**^,^ **^2^, Barbara Beer^1, 2^, André Pick^1^, Teresa Roth^1^, Petra Lommes^1^, Volker Sieber^1^***

^1^ Chair of Chemistry of Biogenic Resources, Straubing Centre of Science, Department Life Science Engineering, Technische Universität München, Straubing, Germany

^2^ Both authors contributed equally

*** Correspondence:** Corresponding Author, Prof. Dr. Volker Sieber, Chair of Chemistry of Biogenic Resources, Straubing Centre of Science, Department Life Science Engineering, Technische Universität München, Schulgasse 16, D-94315 Straubing. [sieber@tum.de](mailto:sieber@tum.de)


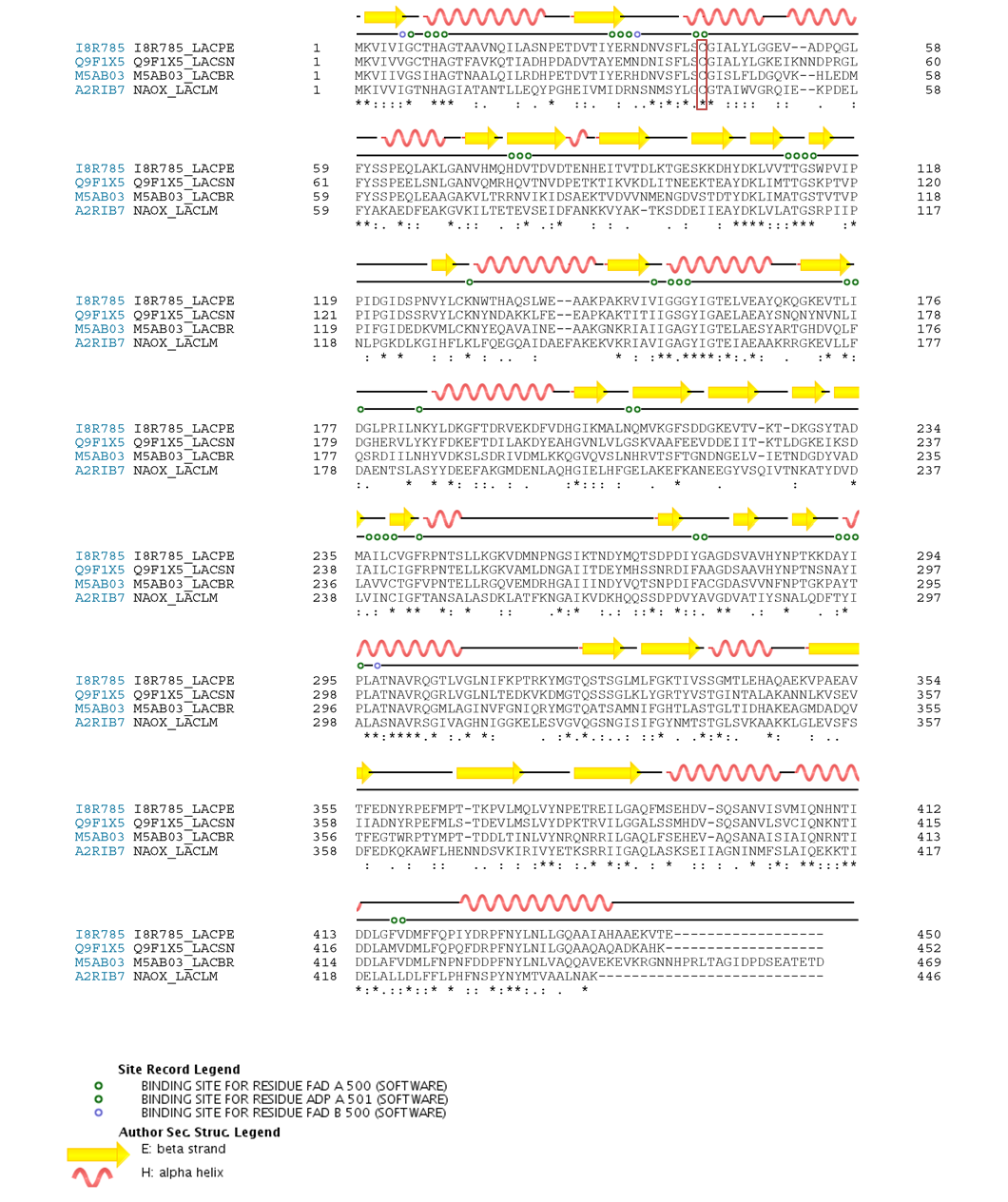


Supplementary Figure 1. Multiple sequence alignment of NADH oxidases. Amino acid sequences of *Lactobacillus pentosus* (Uniprot ID I8R785), *Lactobacillus sanfranciscensis* (Uniprot ID Q9F1X5), *Lactobacillus brevis* (Uniprot ID M5AB03), and *Lactococcus lactis* (Uniprot ID A2RIB7) were aligned using Clustal Omega (Sievers et al., 2011). The secondary structure assignment and the binding site for FAD/ADP of Nox from *L. sanfranciscensis* (PDB ID: 2CDU) is depicted above the alignment. The catalytically active Cys42 is marked in a red box.


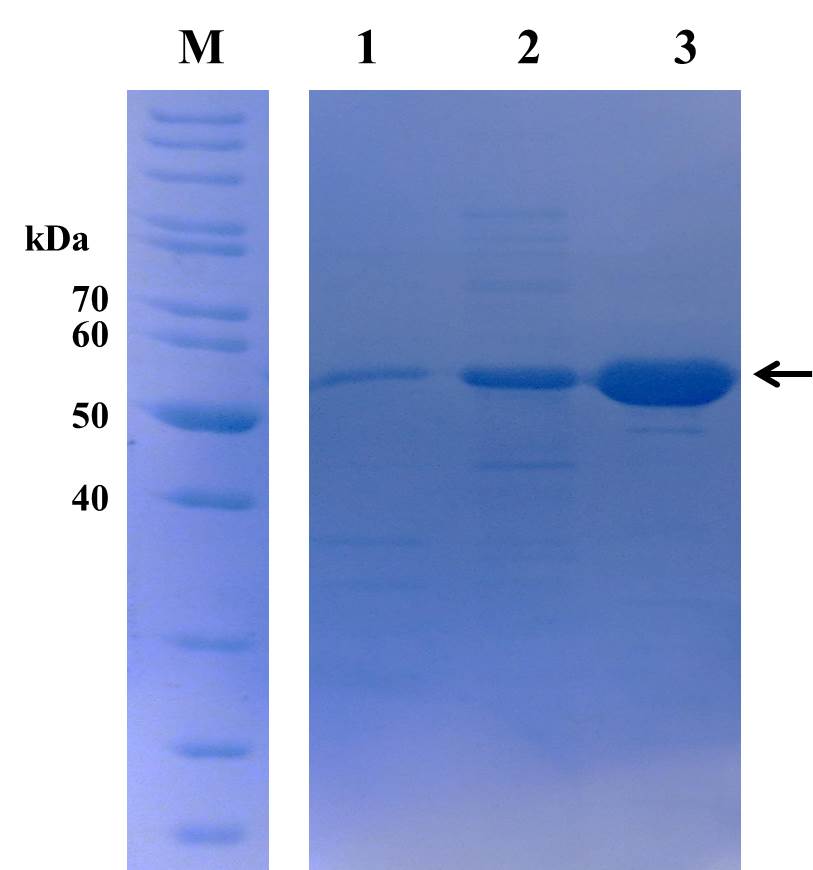


Supplementary Figure 2: SDS-PAGE of *Lp*Nox purification. Lane 1: insoluble fraction of the cell lysate, lane 2: soluble fraction of the cell lysate, lane 3: *Lp*Nox after purification. *Lp*Nox (arrow) is mainly found in the soluble fraction of the lysed cells. *Lp*Nox could be purified to >95 % homogeneity.

**References**

Sievers, F., Wilm, A., Dineen, D., Gibson, T.J., Karplus, K., Li, W., Lopez, R., Mcwilliam, H., Remmert, M., Söding, J., Thompson, J.D., and Higgins, D.G. (2011). Fast, scalable generation of high-quality protein multiple sequence alignments using Clustal Omega. *Molecular Systems Biology* 7**,** 539-539. doi: 10.1038/msb.2011.75.
